# Supplementary material for: Sec20 Is Required for Autophagic and Endocytic Degradation Independent of Golgi-ER Retrograde Transport
Source: Cells. 2019 Jul 24;8(8):768. doi: 10.3390/cells8080768 (PMC6721519; doi:10.3390/cells8080768)
Supplement: Supplementary file 1 [file cells-08-00768-s001.pdf]

cells

MDPI

Supplementary Materials

Figure S1. Sec20 is responsible for proper endocytic trafficking and Golgi structure.

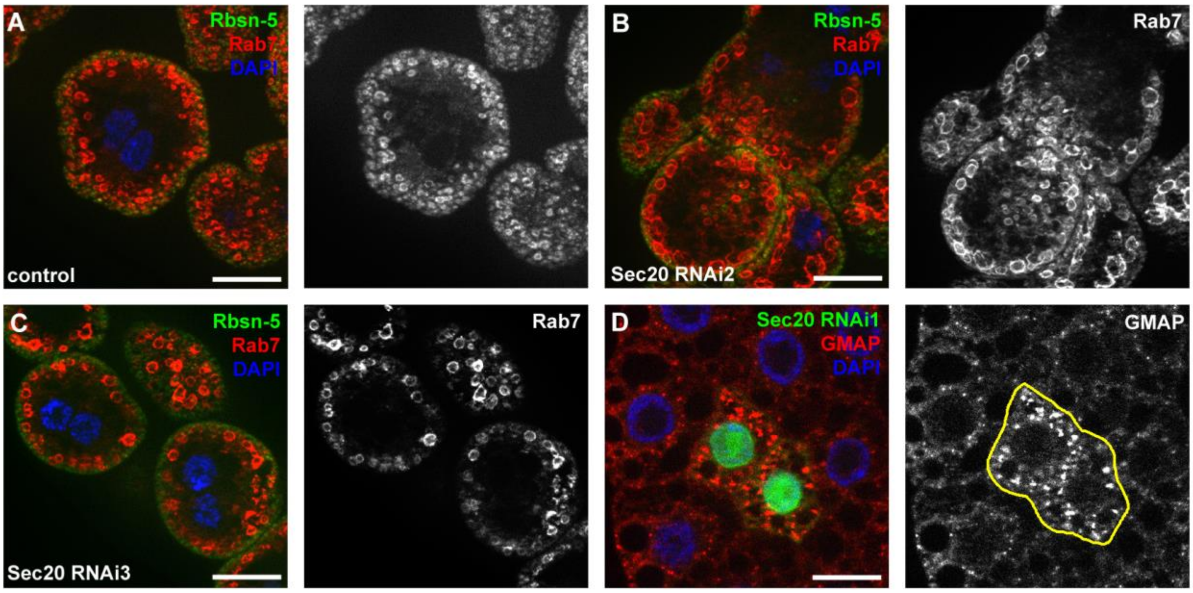

(**A-C**) The knockdown of Sec20 using another two independent RNAi lines in garland nephrocytes results in the enlargement of Rab7-positive late endosomes, similar to the RNAi line shown in Figure 1B. The quantification of these experiments is shown in Figure 1G. (**D**) Anti-GMAP immunostaining shows the accumulation of Golgi compartments in GFP+ Sec20 RNAi fat cells compared to neighboring control cells. Scale bars: 20 μm in panels **A-C**, 10 μm in panel **D.**

Figure S2. Normal early endosomal size and impaired lysosomal degradation in Sec20-depleted nephrocytes.

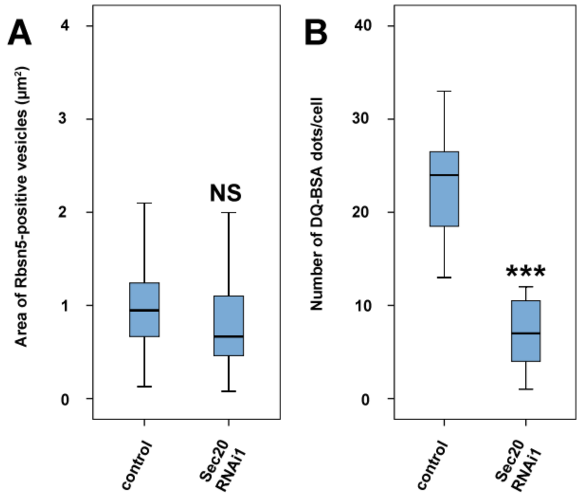

(**A**) Quantification of Rbsn5-positive early endosomal size of nephrocytes displayed on Figure 1 in control and Sec20 RNAi cases. (**B**) Quantification of the number of DQ-BSA puncta in control and Sec20 RNAi nephrocytes after 5 minutes uptake and 30 minutes chase of DQ-BSA. Medians are shown as horizontal black lines within the boxes. Bars show the upper and lower quartiles, and the whiskers plot the smallest and largest observations. NS: non-significant ($p >= 0.05$), ***: $p<0.001$

Figure S3. Endoplasmic reticle is highly enlarged in Sec20 RNAi cells.

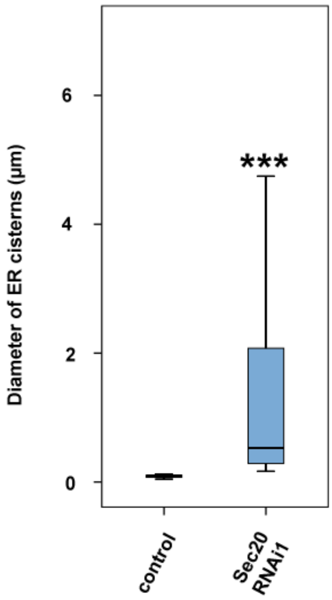

Quantification of the size of ER cisterns in fat cells. The diameter of each individual ER cistern was measured in control and Sec20 depleted cells. Medians are shown as horizontal black lines within the boxes. Bars show the upper and lower quartiles, and the whiskers plot the smallest and largest observations. ***: $p<0.001$

Figure S4. Sec20 and its known partners are all essential for Glue granule production in salivary glands.

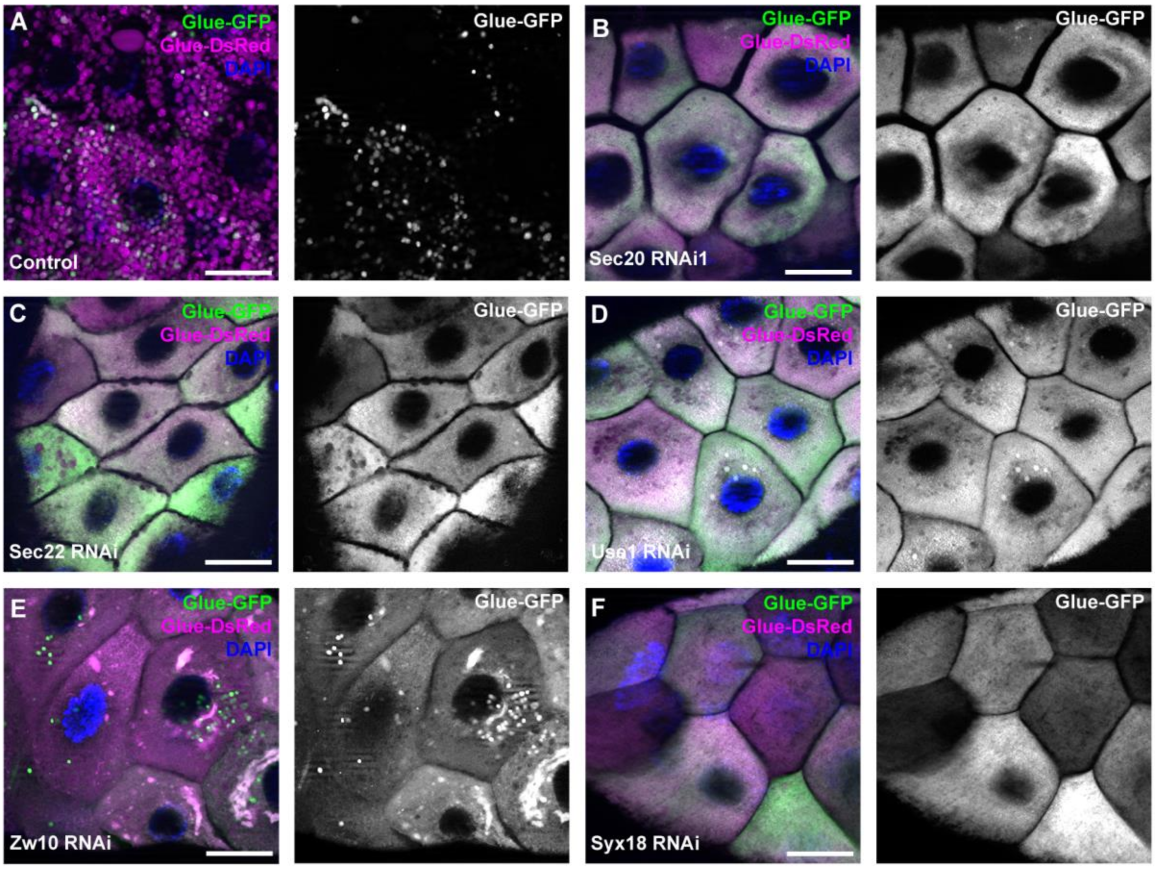

(**A-G**) Salivary glands expressing Glue-DsRed and Glue-GFP of control (**A**) or RNAi flies at the time of puparium formation. While Glue granules properly form and are degraded in control cells (as the pH-sensitive GFP is quenched in the majority of the granules), granules are mostly absent in all knockdown cells. Scale bars: 10 µm.
